# Epigenetic regulation of *JASMONATE ZIM-DOMAIN* genes contributes to heat tolerance in the heat-tolerant rice cultivar Nagina 22

**DOI:** 10.1007/s42994-025-00229-0

**Published:** 2025-07-16

**Authors:** Xiaoxuan Du, Yingnan Sun, Yonggang He, Haiya Cai, Xiangsong Chen

**Affiliations:** 1 State Key Laboratory of Hybrid Rice, College of Life Sciences, Wuhan University, Wuhan, 430072 China; 2 Hubei Hongshan Laboratory, Wuhan, 430070 China; 3 Hubei Key Laboratory of Food Crop Germplasm and Genetic Improvement, Key Laboratory of Crop Molecular Breeding, Ministry of Agriculture and Rural Affairs, Food Crops Institute, Hubei Academy of Agricultural Sciences, Wuhan, 430064 China; 4 Scientific Observation and Experiment Station for Crop Gene Resources and Germplasm Enhancement in Hubei, Ministry of Agriculture and Rural Affairs, Wuhan, 430064 China

**Keywords:** Heat, Nagina 22, JAZ, DNA methylation

## Abstract

Extreme hot weather severely limits rice (*Oryza sativa*) production. Rice cultivars from regions with hot weather are a valuable resource for breeding heat-tolerant rice, but the mechanisms mediating heat tolerance in these cultivars are not fully understood. Here, we investigated heat-tolerance mechanisms in rice using the well-known heat-tolerant cultivar Nagina 22 (N22) and comparing it with the less heat-tolerant cultivar 93–11. Following heat shock (HS) treatment (45 °C for 3 or 24 h), the expression of *JASMONATE ZIM-DOMAIN* (*JAZ*) genes spiked during the early stages of HS responses in N22 but not 93–11 and genes related to jasmonic acid (JA) signaling were repressed in N22. Promoting JA signaling in N22 via pretreatment with methyl JA (MeJA) impaired the heat tolerance of N22, measured as survival after HS treatment of 45 °C for 30 h, followed by a 7-d recovery. Furthermore, the N22-specific activation of *JAZ* genes was associated with increased histone acetylation and decreased DNA methylation. Comparing N22 to 93–11, we propose that the DNA demethylation process, rather than the hypomethylation status per se, is likely associated with *JAZ* activation. In summary, we revealed epigenetic mechanisms that may contribute to the heat tolerance of N22 via the JA signaling pathway; our findings have implications for improving heat tolerance in rice and other crops.

**Supplementary Information:**

The online version contains supplementary material available at 10.1007/s42994-025-00229-0.

## Introduction

Global warming coupled with extreme hot weather events has severely damaged crops (Lobell et al. 2011; Peng et al. 2004). The optimal growth of rice (*Oryza sativa*) is in the summer, making it particularly vulnerable to hot weather. Developing heat-tolerant rice cultivars may limit damage due to hot weather events, and thus help secure food production. Heat-tolerant rice germplasm provides an excellent resource for understanding heat-tolerance mechanisms and cloning heat-tolerance genes (Liu et al. 2023). For example, the heat-tolerance genes *THERMO-TOLERANCE 1* (*TT1*), *TT2*, and *TT3.1*/*TT3.2* were identified in African rice varieties through quantitative trait locus (QTL) mapping (Kan et al. 2022; Li et al. 2015; Zhang et al. 2022). However, the heat-tolerance genes identified to date are not sufficient for crop improvement, as high temperatures affect all cellular activities. In addition, many of the heat-response and heat-tolerance mechanisms in rice remain unclear, which restricts the development of heat-tolerant rice.

Plant hormones are involved in plant development and stress responses; for example, jasmonic acid (JA) and its derivatives are important players in plant responses to environmental stress, such as mechanical damage and insect infestation (Howe et al. 2018). Overexpressing *JASMONATE ZIM-DOMAIN* (*JAZ*) genes increases heat tolerance in rice (Wu et al. 2022). JAZ proteins inhibit JA signaling by binding to MYC transcription factors and preventing them from activating genes involved in JA-related responses. In Arabidopsis (*Arabidopsis thaliana*), defects in JA biosynthesis enhance tolerance of combined heat and cadmium stress (Oshita et al. 2023). Notably, disrupting JA biosynthesis negatively affects heat tolerance in *Arabidopsis* and eggplant (*Solanum melongena*) (Balfagon et al. 2019; Guo et al. 2024; Liu et al. 2024). However, the role of JA in the plant response to heat remains to be fully elucidated.

Epigenetic modifications play important roles in plant stress responses (Chang et al. 2020; Xue et al. 2025; Xie and Duan 2023; Zhang et al. 2024; Zhang and Zhu 2025), including plant responses to heat stress (Chang et al. 2020; Lamke et al. 2016; Popova et al. 2013). Heat stress causes global changes in chromatin modifications, which tend to make the chromatin more accessible (Yang et al. 2023). Although the functions and regulatory mechanisms of heat stress–induced open chromatin are not fully understood, such chromatin may enable the quick activation of heat stress–responsive genes.

In this study, we performed multi-omics analysis to study the mechanisms of heat tolerance in the well-known heat-tolerant rice cultivar Nagina 22 (N22). We show that *JAZ* genes are specifically upregulated by HS in N22, which may contribute to its heat tolerance by repressing JA signaling. We also assessed the effects of JA on heat tolerance in rice. Finally, we examined genome-wide chromatin modifications in rice under heat stress and investigated the roles of these modifications in regulating *JAZ* gene expression during heat-stress responses. Our findings shed light on the roles of epigenetic regulation of JA signaling in heat tolerance in rice, with implications for improving stress tolerance in grain crops.

## Results

### JA signaling is impaired in N22 upon HS

**Fig. 1:**
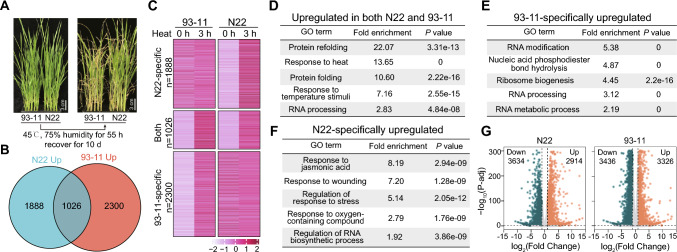
JA signaling-related genes are predominately upregulated in N22 upon HS. **A** Phenotypes of N22 and 93–11 rice seedlings after HS treatment. The plants were treated at 45 °C (75% relative humidity) for 55 h, followed by 10 days of recovery. **B** Venn diagram showing the number of overlapping HS-upregulated genes between N22 and 93–11. **C** Heatmap showing the expression patterns of genes in each group shown in B. **D**–**F** GO analysis of genes that were upregulated in both N22 and 93–11 (**D**) or specifically in N22 (**F**) or 93–11 (**E**) after HS treatment. The top five most highly enriched pathways are listed for each group of genes. **G** Volcano plots showing differentially expressed genes after 3 h of HS treatment in N22 and 93–11

To explore heat-tolerance mechanisms in rice, we examined the well-known heat-tolerant rice cultivar (*Oryza sativa* L. ssp. *indica*/*xian*) Nagina 22 (N22) and compared it with the less heat-tolerant *indica*/*xian* cultivar 93–11 (Fig. 1A). We treated both cultivars with heat shock (HS; 45 °C) for 3 or 24 h and then performed transcriptomic analysis (Fig. S1A and S1B). Known HS-responsive genes (*OsHSP17.0*, *OsHSFA3*, *OsHSP18.0-CII*) were strongly upregulated by HS treatment in N22 and 93–11 (Fig. S1C), confirming the success of the treatments.

To capture the early response to HS, we focused on differentially expressed genes at 3 h of treatment. We identified 2,914 and 3,326 genes upregulated by HS in N22 and 93–11, respectively, with a cutoff of fold change ≥ 2 and *P*-adj ≤ 0.05 (Fig. 1G, Table S1), including 1026 genes upregulated in both N22 and 93–11 (Fig. 1B and C). Gene Ontology (GO) analysis showed that these overlapping genes were highly enriched in heat-responsive pathways, such as response to heat, protein folding, and protein refolding (Fig. 1D), suggesting that heat-responsive pathways were activated in both N22 and 93–11. We then examined the genes that were specifically upregulated in N22 or 93–11. JA-related pathways were predominantly enriched in N22 (Fig. 1F), whereas RNA biogenesis and metabolic pathways, and ribosome biogenesis pathways were enriched in 93–11 (Fig. 1E). JA is a stress-related plant hormone (Howe et al. 2018) and the energy-intensive process of ribosome biogenesis is usually suppressed in response to stress. Therefore, the upregulated JA-related pathways might contribute to the heat tolerance of N22.

### JA signaling is repressed in N22 upon HS

**Fig. 2:**
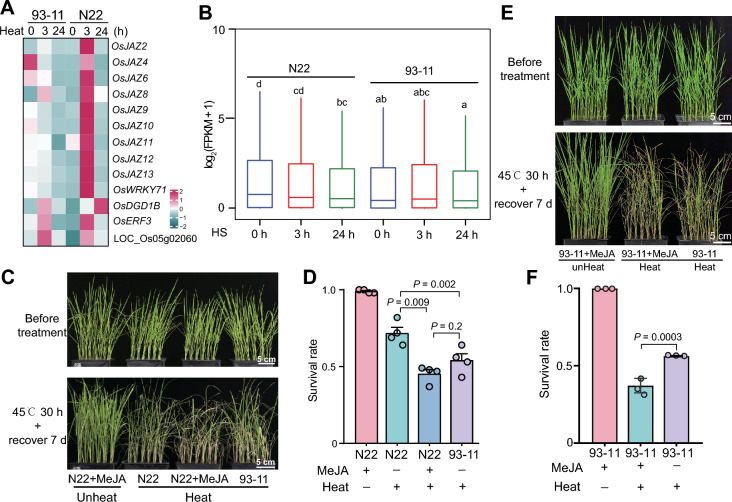
Upregulation of *JAZ* genes contributes to the HS tolerance of N22. **A** Heatmap showing the expression patterns of JA-responsive genes enriched in N22. **B** Boxplot showing the expression levels of JA-responsive genes in N22 and 93–11 during HS treatment. FPKM, fragments per kilobase of exon model per million mapped reads. **C** HS phenotypes of N22 pretreated with MeJA. **D** Survival rates of plants in (C). Data are presented as mean ± SEM of 4 biological replicates. At least 40 individuals were used per replicate. *P* values were calculated by *t*-test. **E** HS phenotypes of 93–11 pretreated with MeJA. **F** Survival rates of plants in (E). Data are presented as mean ± SEM of 3 biological replicates. At least 40 individuals were used per replicate. *P* values were calculated by *t*-test

The abovementioned data prompted us to examine the roles of JA signaling in the heat tolerance of N22. We examined the genes in the “response to jasmonic acid” GO term that were upregulated in N22, finding that 9 out of 13 encoded JAZ proteins (Fig. 2A). Notably, the expression of *JAZ* genes spiked at 3 h of HS treatment and returned to normal levels after 24 h of treatment in N22, whereas they showed steady expression in 93–11 during HS treatment (Fig. 2A). Overexpressing *JAZ* genes enhances heat tolerance in rice (Wu et al. 2022), suggesting that the N22-specific upregulation of *JAZ*s might contribute to its heat tolerance.

JAZ proteins negatively regulate the expression of JA-targeting genes by inhibiting the activities of MYC transcription factors (Zhang et al. 2015). We therefore identified JA-targeting genes by examining the upregulated genes in *MYC*-overexpressing plants (Uji et al. 2016) and assessed the expression of these genes in N22. Consistent with the upregulation of *JAZs*, JA-targeting genes were significantly downregulated in N22 after 3 h of HS treatment (Fig. 2B and Table S2). Notably, although the expression of *JAZs* returned to normal levels after 24 h of HS, the expression of JA-targeting genes continued to be repressed (Fig. 2B and Table S2). By contrast, the expression of JA-targeting genes was not significantly altered in 93–11 during HS treatment (Fig. 2B and Table S2). These data demonstrate that under HS, JA signaling is repressed in N22 but is not significantly affected in 93–11.

### JA negatively regulates the heat tolerance of N22

We then examined whether repressed JA signaling contributes to the heat tolerance of N22. To this end, we pretreated N22 seedlings with MeJA, removed the MeJA, and immediately performed HS treatment (45 °C for 30 h, followed by a 7-d recovery). Without MeJA pretreatment, N22 showed significantly higher survival rates than 93–11 under HS treatment (Fig. 2C and D). However, pretreatment with MeJA reduced the survival rate of N22 to a level similar to that of untreated 93–11 (Fig. 2C and D), indicating that enhanced JA signaling impairs the heat tolerance of N22. MeJA treatment also decreased the survival rate of 93–11 after HS treatment (Fig. 2E and F), demonstrating the role of JA in regulating heat tolerance. These data suggest that N22 acquires heat tolerance at least partially through repressing JA signaling by upregulating *JAZ* genes early in HS responses.

### HS induces dynamic changes in histone acetylation and chromatin accessibility at *JAZ* genes in N22

**Fig. 3:**
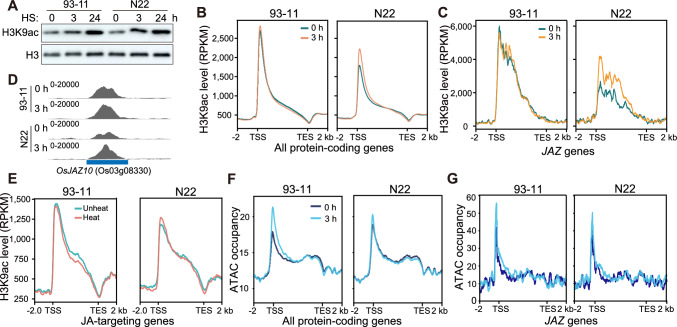
HS causes elevated H3K9ac and increased chromatin accessibility.** A** Immunoblots showing H3K9ac levels in 93–11 and N22 under HS. **B** Metaplots showing H3K9ac levels on all protein-coding genes in 93–11 and N22 under HS. RPKM, reads per kilobase per million mapped reads; TSS, transcription start site; TES, transcription end site; − 2 and 2 kb represent 2 kilobases upstream of the TSS and downstream of the TES, respectively. **C** Metaplots showing H3K9ac levels on *JAZ* genes in 93–11 and N22 under HS. **D** IGV view of H3K9ac levels on a representative *JAZ* gene. **E** Metaplots showing H3K9ac levels on JA-targeting genes under HS. **F** Metaplots showing ATAC signals on all protein-coding genes in 93–11 and N22 under HS. **G** Metaplots showing ATAC signals on *JAZ* genes in 93–11 and N22 under HS

Histone acetylation is a hallmark of gene activation. We therefore investigated whether histone acetylation is involved in regulating *JAZ* gene expression. We first performed immunoblotting for the key epigenetic mark histone H3 lysine 9 acetylation (H3K9ac). Overall, H3K9ac levels increased in response to HS in both N22 and 93–11 (Fig. 3A and S2A). To explore this issue in more detail, we performed H3K9ac ChIP-seq of N22 and 93–11 under normal conditions and at 3 h of HS. We observed higher basal H3K9ac levels on genes in 93–11 than in N22 under normal conditions. After 3 h of HS treatment, the H3K9ac level showed little change in 93–11 but increased slightly in N22 (Fig. 3B). We then examined the H3K9ac levels at *JAZ* genes and JA-responsive genes. In line with the overall tendency, H3K9ac levels at *JAZ* genes increased in N22 after HS treatment but remained steady in 93–11 (Fig. 3C and D and S2B). By contrast, the H3K9ac of JA-responsive genes was not significantly altered (Fig. 3E). Taken together, these findings suggest that dynamic histone acetylation levels during HS might be involved in regulating *JAZ* genes in N22.

Histone acetylation is often associated with open chromatin. To reveal the chromatin environment at *JAZ* loci, we evaluated chromatin accessibility by performing Assay for Transposase-Accessible Chromatin coupled with high throughput sequencing (ATAC-seq). Overall, ATAC signals were substantially higher at the transcription start sites of genes after 3 h of HS treatment in 93–11 but only slightly higher than those before treatment in N22 (Fig. 3F and S2C). We then examined the chromatin accessibility of *JAZ* genes, finding that the ATAC signals of *JAZs* were also higher after 3 h of HS treatment (Fig. 3G). Surprisingly, although the expression levels of *JAZs* were not significantly altered in 93–11, the ATAC signal was consistently higher during HS treatment than before treatment in 93–11 (Fig. 3C), suggesting that chromatin accessibility is not the major factor regulating *JAZ* gene expression under HS. We then examined the ATAC signals of JA-responsive genes during HS treatment. Consistent with their expression patterns, the ATAC signals significantly decreased in N22 but increased in 93–11 upon HS treatment (Fig. S2D).

### N22 displays more variation in DNA methylation after HS

**Fig. 4:**
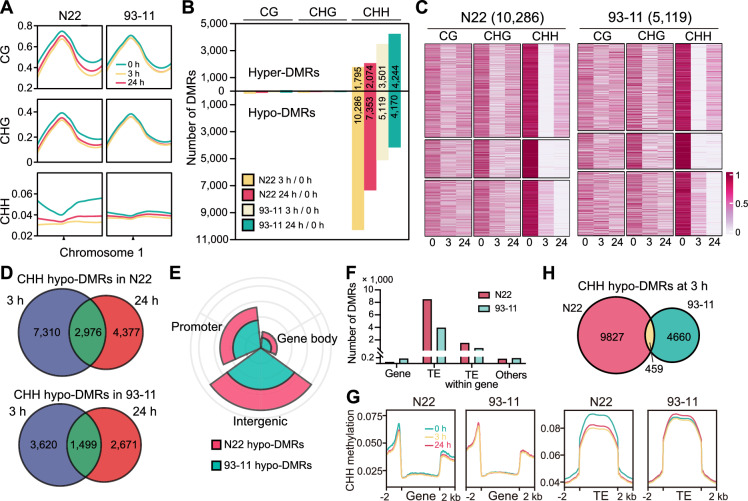
DNA methylation is mainly reduced on TEs in N22 and 93–11 under HS.** A** Chromosomal view of changes in DNA methylation in 93–11 and N22. Data for chromosome 1 are shown. **B** DMRs identified in 93–11 and N22 under HS treatment. **C** Heatmaps showing the methylation levels on CHH hypo-DMRs at 3 h of HS treatment. **D** Venn diagrams showing the overlap of CHH hypo-DMRs between 3 and 24 h of HS treatment in 93–11 and N22. **E** Pie diagram showing the genomic distribution of CHH hypo-DMRs at 3 h of HS treatment in 93–11 and N22. **F** Distribution of hypo-DMRs at 3 h of HS treatment on genes and TEs. **G** Metaplots showing CHH methylation levels on genes and TEs during HS treatment in 93–11 and N22. **H** Venn diagram showing the overlap of CHH hypo-DMRs between 93 and 11 and N22 at 3 h of HS treatment

To further investigate how *JAZ* genes are regulated in N22 and 93–11, we examined DNA methylation by performing whole-genome bisulfite sequencing. In N22, we observed a global reduction of DNA methylation after 3 h of HS treatment, especially in the CHH context (H = A, T, or C) (Fig. 4A). This result is similar to previous findings in *Arabidopsis* (Yang et al. 2023). After 24 h of HS treatment, CHH methylation recovered slightly (Fig. 4A). By contrast, little global change was observed in 93–11 during HS treatment (Fig. 4A). Notably, the CHH methylation level was substantially higher in N22 than in 93–11 under normal conditions. After long-term (24-h) HS treatment, the overall methylation levels were similar between N22 and 93–11 (Fig. 4A).

We then characterized differentially methylated regions (DMRs) in N22 and 93–11 after HS treatment, finding that almost all DMRs were in the CHH context (Fig. 4B). We therefore focused on CHH methylation. Consistent with the overall trends (Fig. 4A), we identified many more hypo-DMRs (10,286) than hyper-DMRs (1,795) in N22 at 3 h of HS treatment (Fig. 4B). In 93–11, we observed similar numbers of hypo-DMRs (5,119) and hyper-DMRs (3,501) after 3 h of HS treatment (Fig. 4B), which could explain why the overall CHH methylation level in 93–11 was not significantly altered. Notably, approximately half of the hypo-DMRs at 3 h of HS treatment recovered to a great extent, and approximately 1/3 showed further demethylation after 24 h of HS treatment (Fig. 4C). Consistent with this, hypo-DMRs at 3 and 24 h of treatment did not strongly overlap (Fig. 4D), indicating that CHH methylation is highly dynamic during HS.

We then explored the relationship between DMRs in N22 and 93–11. To capture the early response of CHH methylation to HS, we focused on hypo-DMRs at 3 h of HS treatment. We observed similar patterns of genomic distribution in N22 and 93–11: most DMRs were located in intergenic regions, and some were located in promoter regions (Fig. 4E). Since transposable elements (TEs) are heavily methylated, we examined methylation on TEs. Indeed, most DMRs were located in TE regions in both N22 and 93–11, whereas DMRs in 93–11 were more likely to be located on genes than those in N22 (Fig. 4F). Consistent with this, CHH methylation levels on genes were not affected by HS treatment in N22 or 93–11, whereas those on TEs significantly decreased in N22 and showed little change in 93–11 during HS treatment (Fig. 4G). We then compared the DMRs in N22 and 93–11, which showed little overlap (Fig. 4H). These data suggest that CHH methylation is affected in different genomic regions between N22 and 93–11.

### HS causes DNA demethylation on MITEs adjacent to* JAZ* genes in N22

**Fig. 5:**
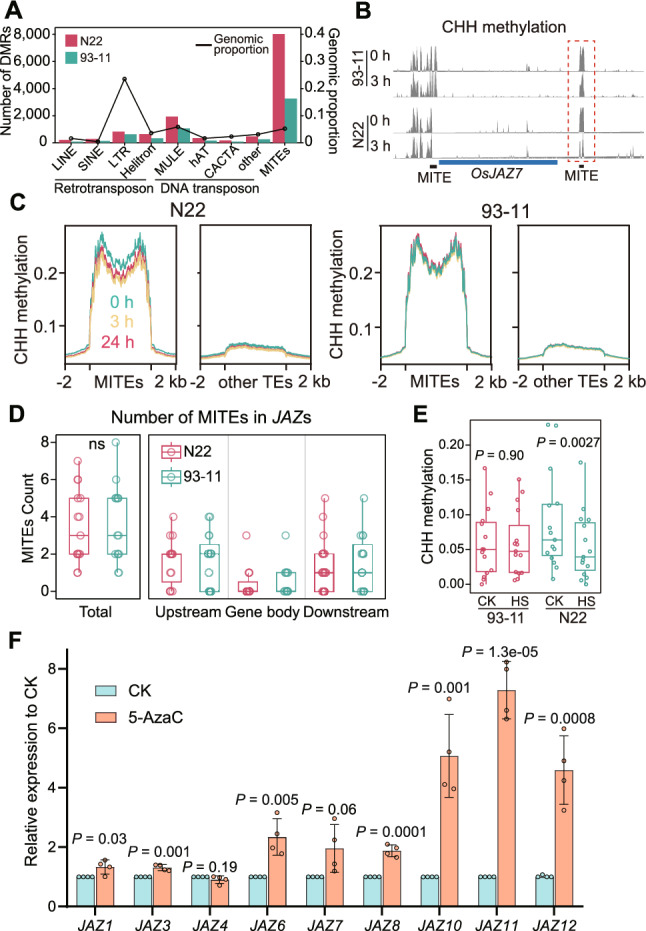
Changes in DNA methylation on MITEs in N22 and 93–11 under HS. **A** Distribution of CHH hypo-DMRs at 3 h of HS treatment on different types of TEs. Physical lengths of the proportions of genomic regions occupied by the respective TEs were used as controls. **B** IGV view of CHH methylation on MITEs surrounding a representative *JAZ* gene upon HS treatment. Dashed box indicates methylation on MITEs. **C** Metaplots showing CHH methylation on MITEs under HS treatment in 93–11 and N22. Other TEs were used as controls. **D** Boxplots showing the number of MITEs adjacent to *JAZ* genes. MITEs within gene bodies and those in regions 2 kb upstream or downstream of *JAZ* genes were counted; ns, no significance. **E** Boxplot showing CHH methylation levels on MITEs adjacent to *JAZ* genes under normal conditions and at 3 h of HS treatment. **F** Expression levels of *JAZ* genes in N22 treated with 5-AzaC. *P* values were calculated by *t*-test. Data are presented as mean ± SEM of 4 biological replicates. *P* values were calculated by *t*-test

To explore DMRs on TEs in detail, we examined the types of TEs occupied by DMRs. Most hypo-DMRs were located on miniature inverted-repeat transposable elements (MITEs) (Fig. 5A). Consistent with this, CHH methylation levels were significantly reduced by HS treatment on MITEs but not on other TEs in N22 (Fig. 5C). MITEs are considered to be truncated derivatives of autonomous DNA TEs (Feschotte and Mouches 2000) that play important roles in regulating gene expression in rice (Lu et al. 2012). We therefore hypothesized that MITEs might regulate *JAZ* genes in N22. To test that idea, we looked for MITEs surrounding *JAZ* genes*.* Indeed, an average of approximately three MITEs surrounded each *JAZ* locus (Fig. 5D and S3A). We then examined the CHH methylation on MITEs adjacent to *JAZs*. CHH methylation on *JAZ*-adjacent MITEs was significantly lower in N22 but unchanged in 93–11 after HS treatment (Fig. 5B, 5E and S3B). Notably, the basal level of CHH methylation before HS was higher in N22 than in 93–11 (Fig. 5E). These observations align with our comparison of N22 and 93–11, which revealed two distinct methylation patterns: under normal conditions N22 exhibited higher global CHH methylation levels compared with 93–11; under HS treatment, N22 exhibited significant attenuation of CHH methylation, whereas 93–11 maintained stable methylation.

Finally, to further assess the role of DNA hypomethylation in heat-induced *JAZ* overexpression in N22, we treated N22 with the DNA methylation inhibitor 5-azacytidine (5-AzaC) under normal conditions (Nowicka et al. 2020) and examined the expression of *JAZ* genes. Most *JAZ* genes were upregulated after 5-AzaC treatment (Fig. 5F), confirming the role of DNA methylation in regulating *JAZ* expression.

## Discussion

In this study, we demonstrated that the N22-specific upregulation of *JAZ* genes coupled with the repression of JA signaling genes may contribute to the heat tolerance of N22. Indeed, treatment with JA decreased the HS tolerance of N22. Consistent with this, a previous study showed that overexpressing *JAZs* enhances heat tolerance in rice (Wu et al. 2022). We further showed that the upregulation of *JAZ*s in N22 is associated with HS-induced DNA demethylation on adjacent MITEs. These findings reveal the roles of JA in the heat tolerance of N22. In addition, they advance our understanding of epigenetic regulatory mechanisms underlying plant stress-tolerance responses.

### Roles of JA in plant heat tolerance

The roles of JA in plant responses to high temperature have attracted increasing attention in recent years (Balfagon et al. 2019; Liu et al. 2024; Oshita et al. 2023; Wu et al. 2022). Given the crucial role of JA in plant defense, it is not surprising that it also plays a key role in plant heat tolerance. However, studies in recent years have reached inconsistent conclusions. For example, overexpressing chloroplast-localized JA biosynthetic genes or exogenous JA treatment enhances heat tolerance in eggplant (Liu et al. 2024), whereas suppressing JA signaling by overexpressing *JAZ* genes enhances heat tolerance in rice (Wu et al. 2022). Even within *Arabidopsis*, JA is reported to have opposite effects on heat tolerance (Balfagon et al. 2019; Oshita et al. 2023). There are several possible reasons for this phenomenon. For instance, in some studies, HS was combined with other stresses, perhaps triggering different intracellular response mechanisms compared with simple HS. In addition, the regulatory mechanisms of JA in response to HS may vary among plant species. The roles of JA in HS responses might also differ at various stages of plant growth. Moreover, although we observed the clear downregulation of JA-responsive genes in N22 but not 93–11 after HS treatment, the absolute expression levels of JA-responsive genes were even lower in 93–11 than in N22 (Fig. 2B), indicating that the downregulation process, rather than the absolute expression levels of JA-responsive genes, might enhance heat tolerance. However, we cannot exclude the possibility that *JAZ* genes regulate heat tolerance through other pathways. A clearer understanding of the underlying mechanism requires further investigation.

### Roles of dynamic chromatin modifications in regulating HS-responsive gene expression

Chromatin modifications, including DNA methylation and histone modifications, play crucial roles in regulating gene expression. Certain chromatin modifications are associated with the activation of gene expression, such as histone acetylation, whereas others are linked to gene silencing, such as DNA methylation. Both our previous and current study indicated that HS induces widespread DNA demethylation and histone hyperacetylation (Yang et al. 2023). The biological significance of this is not entirely clear. One possibility is that extensive chromatin relaxation facilitates the rapid activation of the relevant HS-responsive genes, thereby helping plants survive under sudden HS. We also showed that that in N22, the activation of *JAZ*s is accompanied by an increase in histone acetylation and the removal of DNA methylation, which is not unexpected. Surprisingly, however, in 93–11, although *JAZ* genes showed histone hyper-acetylation and DNA hypomethylation under normal conditions (Fig. 3C and 5E), the expression levels of these genes were not higher in 93–11 than in N22. By contrast, the *JAZ* genes in N22 were in a relatively high state of DNA methylation under normal conditions, yet the reduction in DNA methylation caused by HS treatment was accompanied by the activation of the *JAZ* genes. Therefore, it appears that the process of altering chromatin modifications, rather than the chromatin modifications themselves, is more closely related to changes in gene expression. This finding enriches our understanding of the mechanisms by which chromatin modifications regulate gene expression. Of course, the regulation of gene expression also involves other factors, such as transcription factors, which we did not investigate in this study. Additionally, determining whether this phenomenon is universal requires further investigation.

### Roles of MITEs in plant stress responses

MITEs are abundant in plants, with copy numbers reaching over 170,000 in rice (Lu et al. 2012). Genetic variations of MITEs in the promoter region of a *JAZ* gene in rice can lead to differences in the expression of this gene, thereby affecting heat tolerance (Wu et al. 2022). In the current study, we also showed that genome-wide DNA demethylation under HS conditions mainly occurs in MITEs, which may regulate the expression of *JAZ* genes. Consistent with this, treatment with the DNA methylation inhibitor 5-AzaC increased the expression of *JAZ* genes. Although 5-AzaC may cause DNA demethylation globally in all contexts (CG, CHG, and CHH), these findings collectively suggest that the abundance of MITEs in the rice genome, along with their associated epigenetic modifications such as DNA methylation, plays crucial roles in mediating plant responses to environmental stress. However, how DNA methylation on MITEs senses and responds to stress stimuli requires further study.

## Materials and methods

### Materials and HS treatment

Rice (*Oryza sativa* L. ssp. *Xian*) cultivars 93–11 and Nagina 22 were used in this study. Plants for all experiments were grown in climate-controlled growth chambers. For multi-omics analysis, seeds were planted in soil (potting soil:vermiculite = 2:1) and grown under normal conditions (75% relative humidity; 800 μmol m^−2^ s^−1^ light intensity for 10 h, 30 °C; 14 h of dark, 24 °C) for 18 days. One-third of the plants were harvested as untreated samples (0 h), and the remaining plants were immediately moved to a treatment chamber (75% relative humidity; constant light of 800 μmol m^−2^ s^−1^ intensity; 45 °C). Seedlings without root were harvested after 3 h and 24 h of treatment. For HS treatment phenotyping, plants were grown in nutrient solution (Coolaber Beijing, NSP1040) under normal conditions until 18-day-old, with the solution refreshed every 3 days. The temperature was then increased to 45 °C over the course of 3 h. Treatment at 45 °C was imposed for 55 h after the temperature reached 45 °C. Following treatment, the plants were allowed to recover for 10 days under normal conditions. For JA-HS treatment, plants were grown in nutrient solution until 18-day-old under normal conditions. The solution was refreshed every 3 days. The plants were then transferred to nutrient solution containing 5 μM MeJA and grown under normal conditions for 24 h. The roots were washed with MeJA-free nutrient solution and grown in this solution under HS conditions (75% relative humidity; constant light of 800 μmol m^−2^ s^−1^ light intensity; 45 °C) for 30 h. The plants were then allowed to recover under normal conditions for 7 days.

### RNA isolation, library construction, sequencing, and data analysis

Total RNA was extracted from samples of three biological replicates using Total RNA Extraction Reagent (R401-01, Vazyme). RNA libraries were constructed using a Fast RNA-seq Lib Prep Kit V2 (RK20306, ABclonal). The RNA libraries were sequenced on the DNB-SEQ T7 platform (Annoroad, Beijing, China). For 5-azacytidine treatment, 5-day-old plants were treated with 40 μM 5-AzaC (HY-10586, MedChemExpress) for 7 days and harvested for RNA extraction.

For data analysis, raw paired-end RNA-seq reads were quality-checked using FastQC (v0.11.9) and preprocessed with Trimmomatic (v0.39) (Bolger et al. 2014) to remove adapter sequences and low-quality bases. The cleaned reads were aligned to the *Oryza sativa* reference genome (MSU7.0) using HISAT2 (v2.2.1) (Kim et al. 2019) with default parameters. Differential gene expression analysis was performed using DESeq2 (Love et al. 2014) in R. The raw read counts obtained from StringTie (Shumate et al. 2022) were used as input for DESeq2. The results were visualized using the ComplexHeatmap (Gu et al. 2016) and ggplot2 packages in R. For gene ontology enrichment analysis, a custom GO annotation file was curated for *Oryza sativa* based on the reference genome. Enriched terms were identified using the hypergeometric test, which evaluates the overrepresentation of candidate genes in specific GO categories compared with the genome-wide background. Significance thresholds were set at a false discovery rate (FDR) < 0.05.

### Nuclei extraction and immunoblotting

Approximately 0.5 g finely ground tissue was used for each sample. All protein samples were boiled with 1 × SDS loading buffer at 98  °C for 10 min and separated by 15% SDS-PAGE. The proteins were transferred to PVDF membranes and detected with antibodies against Histone H3 (1:8000, A2348, ABclonal) and H3K9ac (1:5000, A7255, ABclonal). All antibodies were diluted in 1 × TBST buffer containing 3% (w/v) BSA. The immunoblots were developed using the ECL Plus Western Blotting Detection System (21,065, Thermo Fisher Scientific).

### ChIP sequencing and data analysis

Approximately 2 g of plant tissue was ground into a powder in liquid N_2_ and resuspended in 25 mL Nuclear Isolation Buffer (10 mM HEPES pH 8.0, 1 M sucrose, 5 mM KCl, 5 mM MgCl_2_, 0.6% [v/v] Triton X-100, 0.4 mM PMSF, and 1 × Protease Inhibitor Cocktail). Each sample was crosslinked with 1% (v/v) formaldehyde at room temperature for 20 min with rotation, and crosslinking was terminated by adding 1.7 mL 2 M glycine and incubating for 5 min. The homogenate was filtered through a double layer of Miracloth. Following centrifugation at 3000 *g* for 20 min at 4  °C, the pellet was washed with 1 mL ChIP Buffer 2 (10 mM HEPES pH 8.0, 0.25 M sucrose, 10 mM MgCl_2_, 1% [v/v] Triton X-100, 1 mM EDTA, 5 mM β-mercaptoethanol, 1 × Protease Inhibitor Cocktail). The pellet was resuspended in 300 μL Nuclear Lysis Buffer (50 mM Tris–HCl pH 8.0, 10 mM EDTA, 1% [w/v] SDS, 0.1 mM PMSF, 1 × Protease Inhibitor Cocktail) and incubated on ice for 10 min. The sample was then diluted with 700 μL ChIP dilution buffer (1.1% Triton X-100, 1.2 mM EDTA, 16.7 mM Tris–HCl pH 8.0, 167 mM NaCl, 0.1 mM PMSF, and 1 × Protease Inhibitor Cocktail) and sonicated for 15 min (45 s ON/15 s OFF) using a Covaris S220 Focused Ultrasonicator (Thermo Fisher). Following centrifugation for 15 min at 12,000 *g*, the supernatant was incubated overnight with 4 μg anti-H3K9ac antibody (A7255, ABclonal), combined with 40 μL Protein A/G beads (SA032005, Smart-Lifesciences), and incubated for 2 h at 4 °C with rotation. The bead-bound complex was washed three times with 1 mL low-salt buffer (20 mM Tris–HCl 8.0, 150 mM NaCl, 0.1% SDS, 1% Triton X-100, 2 mM EDTA) at 4 °C for 5 min each time. After washing, the complex was released and crosslinking was reversed by boiling at 95 °C for 10 min in 100 μL 10% (w/v) Chelex (1,422,832, BioRad) with shaking at 1000 rpm. The DNA was treated with 2 μL 20 mg/mL Proteinase K and 4 μL 100 μM RNase A and purified for library construction (RK20228, ABclonal) and sequencing (Annoroad).

For data analysis, raw paired-end ChIP-seq reads were aligned to the *Oryza sativa* reference genome using Bowtie2 (v2.3.5) (Langmead and Salzberg 2012) with default parameters. The aligned reads in SAM format were converted to sorted BAM files using SAMtools (v1.9) (Li et al. 2009) with multithreading. Duplicate reads were removed using SAMtools rmdup to generate deduplicated BAM files. To ensure high-confidence alignments, deduplicated reads with a mapping quality (MAPQ) score ≥ 20 were selected by filtering using SAMtools view. The filtered reads were used for downstream peak calling and analysis. Peak regions were identified using MACS2 (v2.2.9.1) (Zhang et al. 2008) with default parameters and a significance threshold of *q*-value < 0.05. Peak genomic locations were annotated using the ChIPseeker package (v1.38.0) in R (Yu et al. 2015).

### ATAC-seq and data analysis

Approximately 0.5 g of each sample material was ground in liquid N_2_, resuspended in 3 mL NE1 Buffer (20 mM HEPES 7.5, 0.5 mM spermidine, 10 mM KCl, 0.5% [v/v] Triton X-100, 20% [v/v] glycerol, 1 × Protease Inhibitor Cocktail), mixed at 4  °C for 10 min, and filtered through two layers of Miracloth. Each sample was then centrifuged at 1800 *g*, 4  °C for 3 min, and the supernatant was discarded. The pellet was washed once with 1 mL NE1 Buffer and twice with 1 mL Wash Buffer (20 mM HEPES pH 7.5, 150 mM NaCl, 0.5 mM spermidine, 1 × Protease Inhibitor Cocktail). The pellet (containing nuclei) was resuspended in 50 μL Tagmentation Buffer (20 mM HEPES 7.5, 0.5 μL 1% [w/v] digitonin, 10 mM MgCl_2_, 0.5 mM spermidine, 1 × Protease Inhibitor Cocktail) and mixed with 5 μL Transposome Mix V50 (12,207, Yeasen) at 37  °C with shaking for 1 h. Samples were treated with 3 μL 20 mg/mL Proteinase K and 1 μL 100 μM RNase A, and these reactions were terminated by adding 10 μL 0.5 M EDTA and incubating at 55  °C for 30 min. DNA was purified and used to construct sequencing libraries using AFTMag NGS DNA Clean Beads (RK20257, ABClonal) and a Hieff NGS Tagment Index Kit for Illumina (12,610, Yeasen). The libraries were sequenced on an Illumina NovaSeq 6000 instrument (Annoroad).

For data analysis, raw paired-end ATAC-seq reads were aligned to the reference genome using Bowtie2 with the parameters –very-sensitive -X 2000. The aligned reads were sorted and converted to BAM format using SAMtools, followed by stringent quality filtering to retain high-confidence alignments (MAPQ ≥ 30). PCR duplicates were removed using Sambamba (v0.8.2) (Tarasov et al. 2015) with –remove-duplicates. Open chromatin regions were identified using MACS3 (v3.0.2) in broad peak mode (–broad) with the parameters –nomodel –shift -100 –extsize 200 to account for transposase cut-site adjustments, and significance was defined as *q*-value < 0.075. Biological replicates were merged by averaging signal intensities. Differentially accessible regions (DARs) were identified using a threshold of *q*-value < 0.05 and |log_2_ fold change|> 1, followed by genomic annotation with ChIPseeker. RPKM (Reads Per Kilobase per Million mapped reads) normalization was applied to the ATAC-seq signals across all samples, ensuring the comparability of chromatin accessibility levels. Additionally, Fraction of Reads in Peaks (FRiP) values were calculated for each sample to assess data quality and potential variation in Tn5 activity; the results are listed in Table S3.

### Whole‐genome bisulfite sequencing and data analysis

Genomic DNA was extracted from approximately 100 mg ground tissue using a DNeasy Plant Mini Kit (69,104, Qiagen). Library construction and sequencing were performed by Annoroad. For data analysis, read alignment and DMR identification were performed as described previously (Yang et al. 2023). BEDtools (Quinlan and Hall 2010) was used to calculate the methylation levels of specific regions.

## Supplementary Information

Below is the link to the electronic supplementary material.

Supplementary file1 Fig. S1 RNA-seq of 93-11 and N22 under HS. A Principal component analysis (PCA) of the RNA-seq data. B Heatmap showing the correlations between replicates of RNA-seq data. C RT-qPCR showing the expression levels of known HS-inducible genes under the treatments conducted in this study. (PDF 612 KB)

Supplementary file2 Fig. S2 Histone acetylation and ATAC-seq of 93-11 and N22 under HS treatment. A Immunoblots showing two additional replicates of H3K9ac levels in 93-11 and N22 under HS. B IGV view of H3K9ac on a representative JAZ gene locus. C Quantification of ATAC-seq peaks. D Boxplots showing ATAC signals on JA-targeting genes. (PDF 665 KB)

Supplementary file3 Fig. S3 Number and DNA methylation of MITEs adjacent to JAZ genes. A Number of MITEs adjacent to JAZ genes. B IGV view of CHH methylation on MITEs surrounding a representative JAZ gene upon HS treatment. Dashed box indicates methylation on MITEs. (PDF 518 KB)

Supplementary file4 (XLSX 1468 KB)

Supplementary file5 (XLSX 456 KB)

Supplementary file6 (XLSX 10 KB)

## Data Availability

High-throughput sequencing data are available at the China National Center for Bioinformation (https://ngdc.cncb.ac.cn/) under accession number PRJCA035812.
