# Supplementary figures and images for: Epigenetic regulation of JASMONATE ZIM-DOMAIN genes contributes to heat tolerance in the heat-tolerant rice cultivar Nagina 22
Source: aBIOTECH. 2025 Jul 16;6(3):441–51. doi: 10.1007/s42994-025-00229-0 (PMC12454248; doi:10.1007/s42994-025-00229-0)

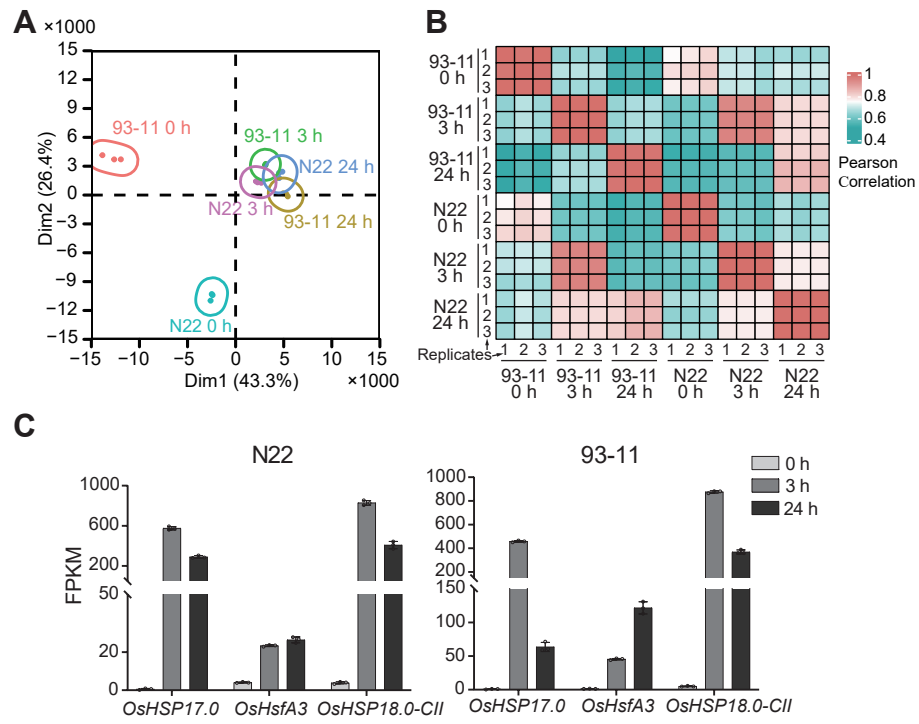

Supplement: Supplementary file 1 — Supplementary file1 Fig. S1 RNA-seq of 93-11 and N22 under HS. A Principal component analysis (PCA) of the RNA-seq data. B Heatmap showing the correlations between replicates of RNA-seq data. C RT-qPCR showing the expression levels of known HS-inducible genes under the treatments conducted in this study. (PDF 612 KB) [file 42994_2025_229_MOESM1_ESM.pdf]

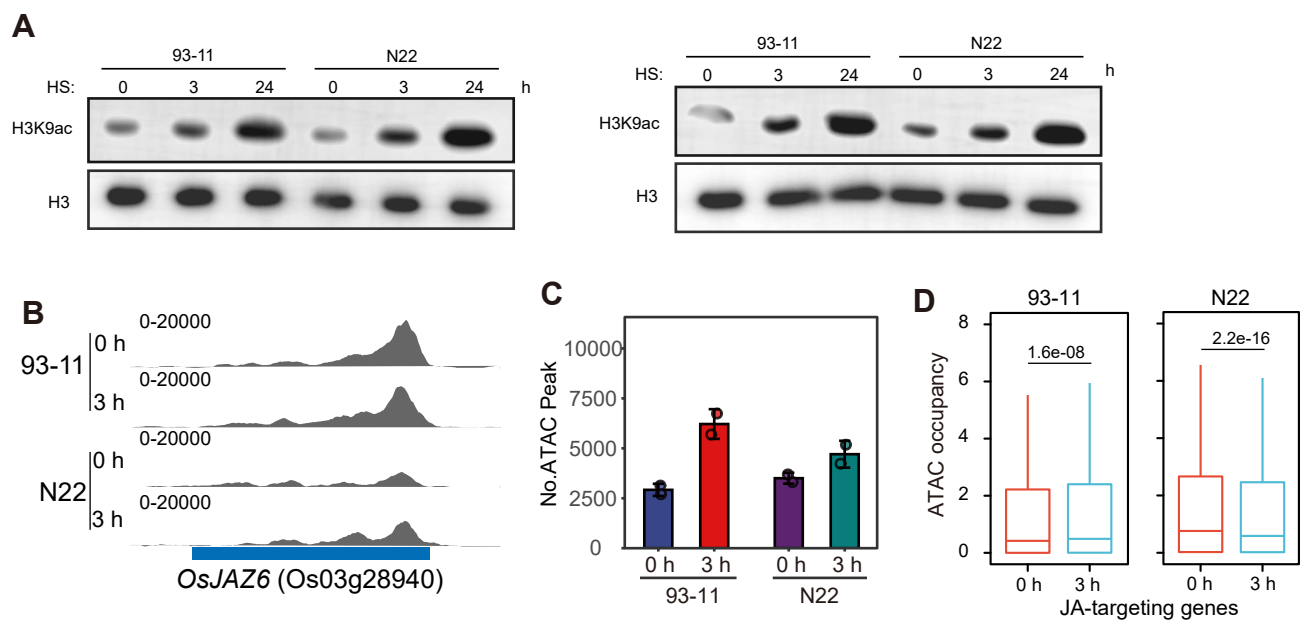

Supplement: Supplementary file 2 — Supplementary file2 Fig. S2 Histone acetylation and ATAC-seq of 93-11 and N22 under HS treatment. A Immunoblots showing two additional replicates of H3K9ac levels in 93-11 and N22 under HS. B IGV view of H3K9ac on a representative JAZ gene locus. C Quantification of ATAC-seq peaks. D Boxplots showing ATAC signals on JA-targeting genes. (PDF 665 KB) [file 42994_2025_229_MOESM2_ESM.pdf]

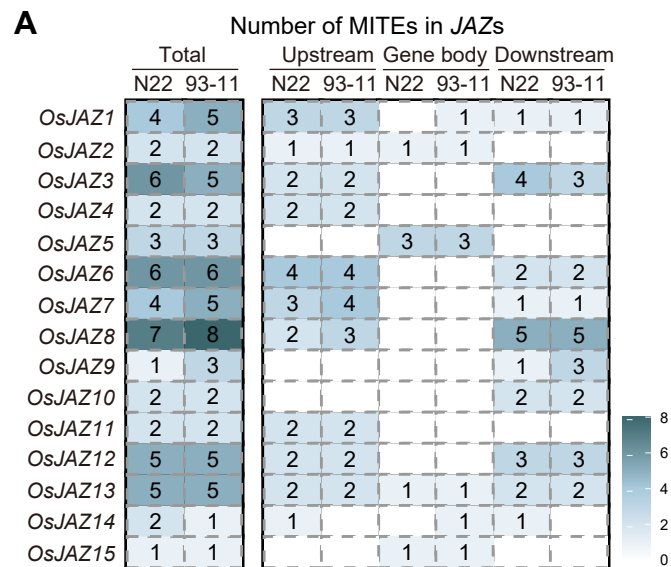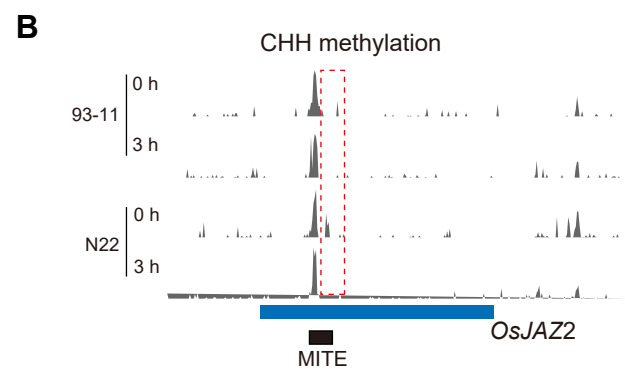

Supplement: Supplementary file 3 — Supplementary file3 Fig. S3 Number and DNA methylation of MITEs adjacent to JAZ genes. A Number of MITEs adjacent to JAZ genes. B IGV view of CHH methylation on MITEs surrounding a representative JAZ gene upon HS treatment. Dashed box indicates methylation on MITEs. (PDF 518 KB) [file 42994_2025_229_MOESM3_ESM.pdf]
